# iSEEtree: interactive explorer for hierarchical data

**DOI:** 10.1093/bioadv/vbaf107

**Published:** 2025-05-06

**Authors:** Giulio Benedetti, Ely Seraidarian, Theotime Pralas, Akewak Jeba, Tuomas Borman, Leo Lahti

**Affiliations:** Department of Computing, Faculty of Technology, University of Turku, Turku FI-20014, Finland; Department of Computing, Faculty of Technology, University of Turku, Turku FI-20014, Finland; Department of Computing, Faculty of Technology, University of Turku, Turku FI-20014, Finland; Department of Computing, Faculty of Technology, University of Turku, Turku FI-20014, Finland; Department of Computing, Faculty of Technology, University of Turku, Turku FI-20014, Finland; Department of Computing, Faculty of Technology, University of Turku, Turku FI-20014, Finland

## Abstract

**Motivation:**

Hierarchical data structures are prevalent across several research fields, as they represent an organized and efficient approach to study complex interconnected systems. Their significance is particularly evident in microbiome analysis, where microbial communities are classified at various taxonomic levels using phylogenetic trees. In light of this trend, the R/Bioconductor community has established a reproducible analytical framework for hierarchical data, which relies on the generic and optimized TreeSummarizedExperiment data container. However, this framework requires basic programming skills.

**Results:**

To reduce the entry requirements, we developed iSEEtree, an R package, which provides a visual interface for the analysis and exploration of TreeSummarizedExperiment objects, thereby expanding the interactive graphics capabilities of related work to hierarchical structures. This way, users can interactively explore several aspects of their data without the need for an extensive knowledge of R programming. We describe how iSEEtree enables the exploration of hierarchical multi-table data and demonstrate its functionality with applications to microbiome analysis.

**Availability and implementation:**

iSEEtree was implemented in the R programming language and is available on Bioconductor at https://bioconductor.org/packages/iSEEtree under an Artistic 2.0 license.

## 1 Introduction

Interactive exploration plays a key role in data science. It supports early analytical stages, such as quality control and hypothesis generation, as well as more advanced ones, such as the interpretation and reporting of the study results. However, the hierarchical nature of data in microbiome research and other fields necessitates specific analytical and visual solutions (Moreno-Indias *et al.* 2021). Therefore, the R/Bioconductor community has developed a robust bioinformatic framework to effectively manage hierarchical data (Gentleman *et al.* 2004; R Core Team 2024). Despite these advances, there remains a considerable gap in the availability of tailored interactive tools to perform exploratory analysis of hierarchical data in fields such as microbiome research.

The current work has been motivated especially by emerging needs in modern microbiome analysis, where data structures are inherently hierarchical. Microbiome data are typically organized in abundance tables, whose rows and columns represent taxonomic features and experimental samples, respectively. Features vary in the degree of relatedness to other features, which leads to a hierarchical structure that is routinely described by phylogenetic trees. Given the complexity of hierarchical systems such as microbial communities, it is critical to carry a diverse and sound toolkit of analytical and visual solutions. To this end, iSEEtree was developed as an R package that combines existing methods for hierarchical data analysis with the interactive graphic capabilities of the iSEE package (Rue-Albrecht *et al.* 2018). This integration enables users to leverage code-based analytical and plotting functionality without the necessity for an extensive programming knowledge thereof.

Previous solutions for microbiome data exploration in R/Bioconductor are either based on the phyloseq or package-specific classes (McMurdie and Holmes 2015; Zhao *et al.* 2021). Instead, iSEEtree relies on the general-purpose data container TreeSummarizedExperiment (TreeSE), and thus it inherits its multi-table functionality and generalizability to a diverse set of hierarchical datasets (Huang *et al.* 2021). In practice, this allows the integration of several assay types in a single data object, which can then be explored with a comprehensive interface where information is actively shared across panels.

Overall, by combining the strengths of existing software for hierarchical data analysis, iSEEtree offers flexible and user-friendly solutions for interactive data visualization and exploration without the need for an extensive knowledge of programming, assisting new and more experienced practitioners in the analysis and discovery of hierarchical multi-table data.

## 2 Software implementation

iSEEtree bridges the gap between effective visualization of hierarchical data and user-friendly interactive analysis. However, several previous packages critically contributed to its development. In particular, this framework relies on the TreeSE data container as its main class and derives its functionality from the mia and iSEE packages available in Bioconductor. Next, we discuss how iSEEtree complements and extends the existing ecosystem.

### 2.1 Data containers

Data containers constitute a standardized and optimized framework to store and manipulate data, ensuring that information is systematically organized and readily accessible. In this context, the TreeSE container is designed to expand the functionality of its superclasses, the SummarizedExperiment and SingleCellExperiment, to hierarchical structures (Huang *et al.* 2021). TreeSE objects can host multiple numeric tables as assays with metadata on samples (columns) and features (rows) as colData and rowData, respectively. Additionally, results from ordination techniques, such as Principal Component Analysis (PCA) and distance-based Redundancy Analysis (dbRDA), can be stored in the reducedDim slot, which was introduced by and is inherited from the SingleCellExperiment class. However, what ultimately distinguishes TreeSE from other data containers lies in its ability to incorporate the hierarchical structures of samples and features in the rowTree and colTree slots, and map them to the assay elements via rowLinks and colLinks, respectively.

The flexibility and self-contained nature of the TreeSE container has led several microbiome analysis frameworks in Bioconductor to adopt it as their base class. For instance, the mia family of packages provide a toolkit to perform transformations, statistical analysis, and visualization on TreeSE objects. Building on this foundation, iSEEtree leverages the strengths of such framework to enable interactive hierarchical data exploration.

### 2.2 Operative framework

**Figure 1.**
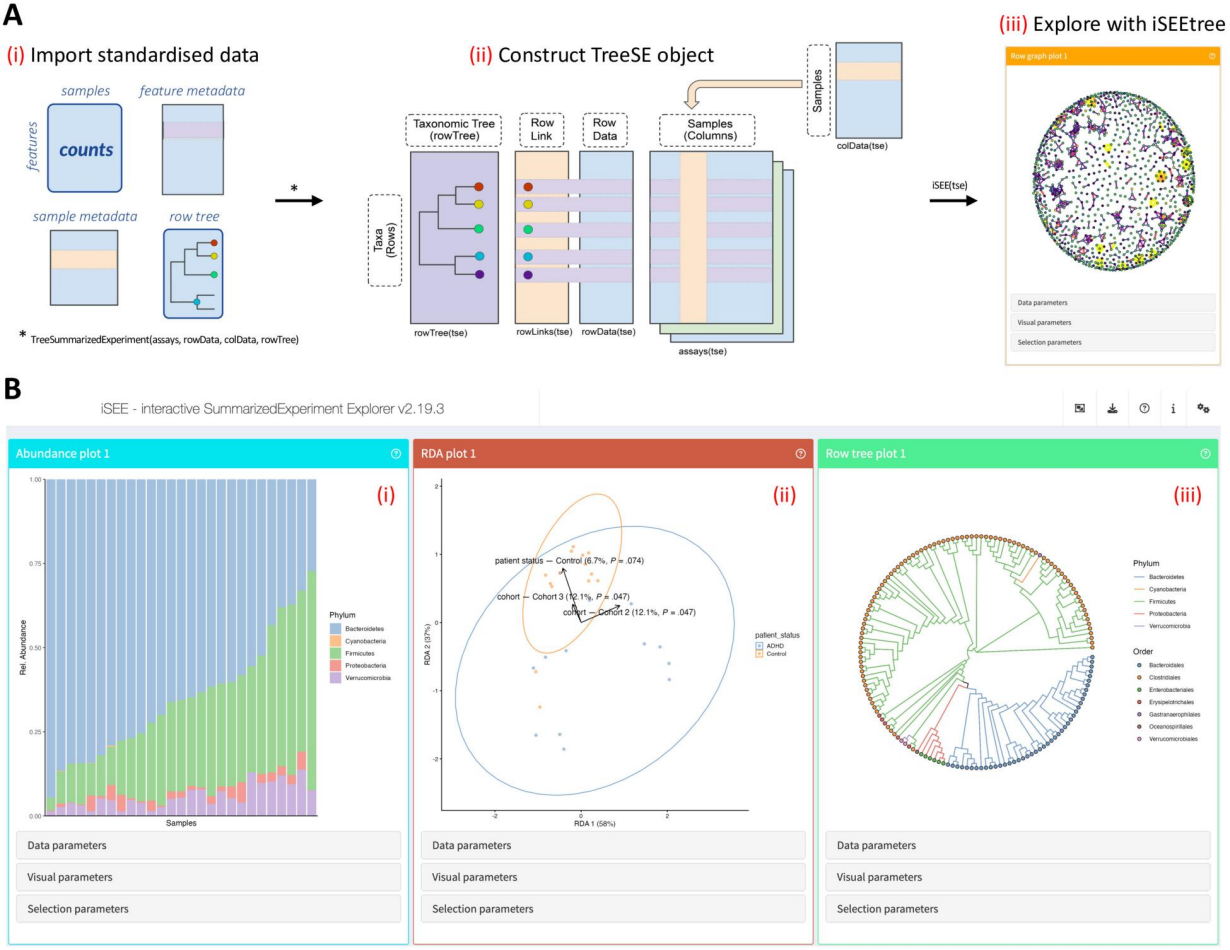
iSEEtree operative framework. (A) Analytical workflow. (i) Data in standardized format are imported in R. This may include abundance assays and optional metadata on features, samples, and tree hierarchies. (ii) A TreeSE data object is constructed from the data. This is an optimized and standardized container for hierarchical data analysis. (iii) The object is input to iSEEtree, which launches an interface with a set of data-dependent visualizations. Adapted from Huang *et al*. (2021). (B) iSEEtree panel layout. The app provides a customizable interface, where each panel describes a certain aspect of the data under investigation. Panels can help explore the sample composition and feature abundance or prevalence (i), identify the main components with ordination analysis (ii) and inspect the hierarchical structure of the data with tree plots (iii). The dataset shown in the snapshot was created from Tengeler *et al*. (2020).

The workflow to use iSEEtree can be divided into three steps (Fig. 1A). First, data are imported from standardized file formats. This may include multiple abundance assays supplemented with metadata on features, samples, and tree hierarchies. Alternatively, ready-to-use datasets are also available to experiment with the app functionality. Next, a TreeSE object is constructed from the data and processed according to one’s visualization needs. This step takes place automatically when using importers for standardized file formats. Finally, the app can be launched from an R console or RStudio by executing the command iSEE(tse), where the input object is of type TreeSE. Otherwise, the panel layout will appear as defined in the parent package iSEE.

Upon launching the app, an interface with a predefined yet customisable layout is created (Fig. 1B). Each panel showcases a certain aspect of the explored dataset and provides several parameters to transform data (*Data parameters*), control aesthetics (*Visual parameters*), and subset the data (*Selection parameters*). Their usage is described in the tours accessible by clicking on the question marks. Once the desired plots are achieved, they can be downloaded to report and publish findings.

### 2.3 App functionality

Currently, iSEEtree provides the tools to perform three different stages of hierarchical data analysis (Table 1). First, preliminary compositional exploration gives an overarching perspective on the structure and organization of the system. Second, supervised and unsupervised ordination techniques estimate diversity across samples and determine the main biological or technical factors that contribute to it. Last but not least, the investigation of hierarchical structures reveals how features or samples relate to one another and can be used to identify dominant groups within the overall system. Such support for hierarchical data is a unique feature of iSEEtree that distinguishes it from other iSEE extensions.

**Table 1.** iSEEtree panel catalogue.^a^

Panel	Information
**Composition**	
AbundancePlot	Feature abundance by sample
AbundanceDensityPlot	Feature distribution across samples
PrevalencePlot	Feature prevalence across samples
FeatureAssayPlot^b^	Feature counts by column variable
ComplexHeatmapPlot^b^	Counts by features and samples
**Ordination**	
RDAPlot	Redundancy Analysis plot
ScreePlot	Explained variance by component
LoadingPlot	Feature loadings by component
ReducedDimensionPlot^b^	Any ordination result
**Structure**	
RowTreePlot	Hierarchical organization of features
RowColumnPlot	Hierarchical organization of samples
RowGraphPlot	Network organization of features
ColumnGraphPlot	Network organization of samples

a Currently, iSEEtree provides 10 panel designs to visualize results from 3 stages of hierarchical data analysis: preliminary compositional exploration, ordination, and structural analysis.

b This panel is inherited from the iSEE parent package.

Our work builds on top of the independently developed package iSEE, which provides panels for the SummarizedExperiment class, a container to organize data in multiple tables supplemented with metadata on samples and features (Rue-Albrecht *et al.* 2018; Huang *et al.* 2021). Consequently, our app inherits the complete set of functionality from this foundation, including multiple interactive panels, selectable parameters, selections across panels, interactive tours, customizable esthetics, code tracking for reproducibility and downloadable results. Panels derived from iSEE are not only supported in our work but also often play a critical role in explorating hierarchical data. Prominent examples include the Feature assay, Complex heatmap, and Reduced dimension plots, as well as the Row and Column data plots and tables.

## 3 Applications

### 3.1 Use cases

The basic usage of iSEEtree is demonstrated in the online package documentation for Tengeler *et al*. (2020), a study on the effects of gut microbiome on attention-deficit/hyperactivity disorder (ADHD) in humanized mice. The dataset contains 151 features from 27 stool samples obtained with 16S rRNA gene sequencing. The tutorial guides users through importing and preparing the dataset for visualisation, launching the app and customizing the initial panel layout. Fig. 1B depicts a snapshot of the app for this dataset containing the abundance, RDA and row tree plots after adjusting their respective parameters. Because those panels can be iteratively modified by a simple interface based on visual feedback, data exploration is more intuitive and less time-consuming compared to the programming alternative. In addition, a selection of samples or features can be simultaneously shared across panels without having to subset the original TreeSE object.

### 3.2 Extensions

Similar visualization approaches for hierarchical data are found across many research fields. However, the preprocessing and analytical methods for such data largely depend on the study objective, which often varies between different fields. In this respect, iSEEtree provides highly generic panels for hierarchical data of any origin, while domain-specific applications can be subsequently developed on its foundation. A notable example of this integration is illustrated by miaDash, a web app for the interactive analysis and exploration of microbiome data. This online extension enables users to import, manipulate, and ultimately visualize microbiome datasets according to their unique requirements, all through a fully graphical interface built on the base of iSEEtree. This way, miaDash compensates for the lack of microbiome-specific operations in iSEEtree, including taxonomic agglomeration and assay transformations. Overall, iSEEtree not only serves as a flexible tool to explore hierarchical data across several domains but it also paves the way for tailored implementations such as the openly available miaDash app.

## 4 Discussion

### 4.1 Limitations

The three major limitations of iSEEtree concern scalability, method coverage, and accessibility. First, the app may significantly slow down as data size increases. Several solutions, mainly derived from the parent iSEE package, are available to optimize performance. It is especially recommended to initialize the interface without unnecessary panels. A more advanced option is to store the assays of the TreeSE object as out-of-memory matrices provided by the HDF5Matrix class, which accesses data from an HDF5 file. When applicable, users can also speed up calculations by reducing the data size (e.g. through subsetting or agglomerating). Second, the current panel catalogue is relatively restricted and parameters do not cover all options available in the corresponding command line environment. Although the R scripts generated by the app can be modified and extended, we also aim to expand the selection of iSEEtree panels and add more interactive features to the existing ones. In particular, interactive functionality typical of shiny apps, such as brushing and hovering to dynamically select points, is desirable for certain operations performed on hierarchical data and thus it will be introduced in future versions. Third, it is still necessary to build TreeSE objects and launch the app either from R or RStudio. As this might represent a barrier for novice users, an entirely graphical interface could be created by combining the generic iSEEtree panels with custom dashboards for domain-specific data analysis. Such approach is illustrated by the miaDash app, which provides an exhaustive toolkit for microbiome data analysis. In summary, greater scalability, method coverage, and accessibility will further improve the app quality and enrich the overall user experience.

### 4.2 Conclusion

Although the study of hierarchical data has become a routine practice in statistical analysis, basic programming skills are typically required to perform even elementary evaluations. To address this need, we developed iSEEtree, an openly available R package that provides a flexible and user-friendly graphical interface to explore hierarchical data. While the app was demonstrated in the context of microbiome data analysis, its functionality is extensible to any domain involving hierarchical data. iSEEtree belongs to the R/Bioconductor ecosystem and benefits from the contributions of the open developer community. By providing a graphical interface with generic panel designs, the app allows new and advanced users, regardless of programming skills, to effectively navigate hierarchical multi-table data and vividly report study results, promoting reproducible practices for scientific discovery in microbiome research and other domains.

## Data Availability

iSEEtree is available under an Artistic 2.0 license on Bioconductor (https://bioconductor.org/packages/iSEEtree). Its usage is further discussed in the online documentation (https://microbiome.github.io/iSEEtree/). This article describes iSEEtree v1.1, which depends on R v4.4 and Bioconductor v3.21.
